# Local excision for anal squamous cell carcinoma: challenges in patient selection and diagnosis

**DOI:** 10.1590/0102-672020260000040e1969

**Published:** 2026-08-31

**Authors:** Lucas Faraco SOBRADO, Giulia Carli MENDES, Caio Sergio Rizkallah NAHAS, Guilherme Cutait de Castro COTTI, Antônio Rocco IMPERIALE, Diego Fernandes Maia SOARES, Carlos Frederico Sparapan MARQUES, Ulysses RIBEIRO

**Affiliations:** 1 Universidade de São Paulo, Faculty of Medicine, Hospital das Clínicas, Instituto do Câncer do Estado de São Paulo, Department of Gastroenterology, Division of Coloproctology – São Paulo (SP), Brazil.

**Keywords:** Chemoradiotherapy, Anus neoplasms, Neoplasm staging, Quimiorradioterapia, Neoplasias do ânus, Estadiamento de neoplasias

## Abstract

**Background:**

Local excision (LE) has a limited role in the management of anal squamous cell carcinoma (SCC), typically restricted to carefully selected early-stage tumors. However, in clinical practice, LE is often performed in broader contexts, particularly in patients with large perianal lesions without prior confirmation of invasive carcinoma, in whom diagnostic uncertainty may influence treatment decisions.

**Aims:**

To evaluate oncologic outcomes after LE for anal SCC, with particular emphasis on the role of lesion characteristics, diagnostic uncertainty, and treatment patterns in real-world practice.

**Methods:**

This was a retrospective study of patients with anal SCC who underwent LE as primary treatment and were followed at a tertiary cancer center between 2010 and 2024. Clinical, pathological, and treatment data were collected, including tumor characteristics, margin status, use of adjuvant therapy, and oncologic outcomes.

**Results:**

A total of 20 patients with invasive anal SCC were included. The mean tumor size was 3.24 cm (standard deviation ±1.75 cm), with lesions up to 6.9 cm. No patients received chemoradiotherapy before LE. Only two patients met conventional criteria for LE (<2 cm, well or moderately differentiated tumors). Overall, 14 patients (70%) required additional treatment following LE, including chemoradiotherapy or abdominoperineal resection. Among patients initially managed with surveillance, 44% developed local recurrence, even in cases with negative margins.

**Conclusions:**

Outcomes following LE for anal SCC are strongly influenced by patient selection and clinical context. In this real-world cohort, most patients outside established criteria required additional treatment, underscoring the limitations of LE in large perianal lesions. These findings highlight the importance of careful preoperative evaluation and maintaining a high index of suspicion for invasive carcinoma to guide appropriate initial management.

## ARTICLE HIGHLIGHTS

Most patients undergoing local excision for anal squamous cell carcinoma did not meet conventional criteria for definitive local treatment.

Large perianal lesions frequently represented a diagnostic challenge, with invasive carcinoma identified only after excision.

Seventy percent of patients required additional treatment after local excision, including chemoradiotherapy or abdominoperineal resection.

Local recurrence occurred even among patients managed with surveillance after negative-margin excision.

Careful preoperative evaluation and high index of suspicion for invasive carcinoma are essential when managing large perianal lesions.

## Summary

**CENTRAL MESSAGE:**

Anal squamous cell carcinoma is an uncommon malignancy whose incidence has increased in recent decades, particularly among immunosuppressed populations. Combined chemoradiotherapy remains the standard of care, providing high rates of local control and survival. Local excision has a limited role and is generally reserved for carefully selected early-stage tumors, particularly small perianal lesions without sphincter involvement. In clinical practice, however, large perianal lesions often present a diagnostic challenge.

**PERSPECTIVES:**

The study demonstrates that outcomes following local excision for anal squamous cell carcinoma are strongly influenced by patient selection and clinical context. In this real-world cohort, most patients outside established criteria for local excision required additional treatment, underscoring the complexity of managing large perianal lesions and the limitations of local excision in this setting. These results support a high index of suspicion for invasive carcinoma in large lesions and emphasize the importance of careful preoperative evaluation to guide optimal initial management.

## VISUAL ABSTRACT

**Figure:**
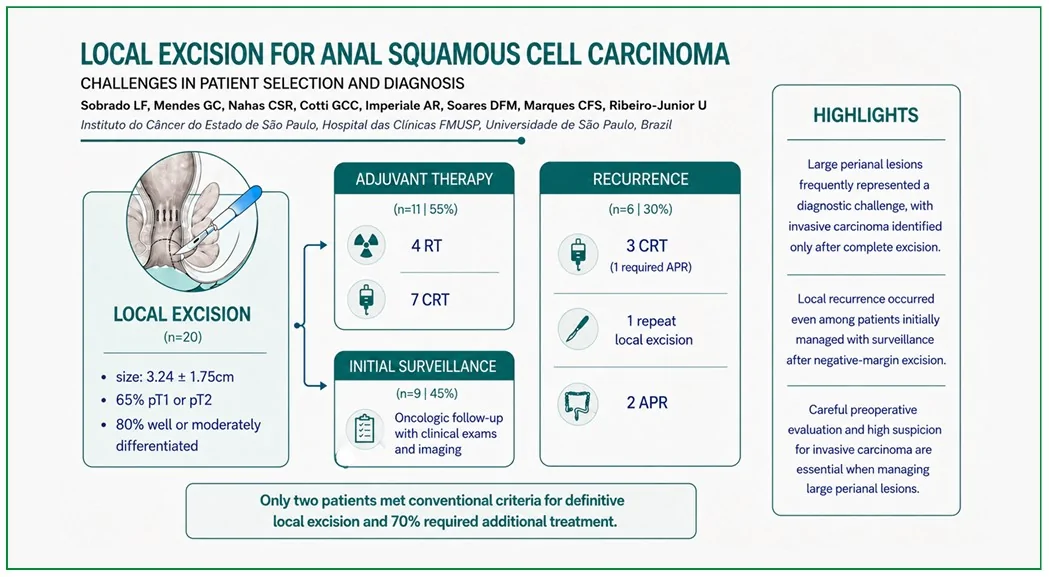

## RESUMO

**Racional:**

A excisão local (EL) possui papel limitado no manejo do carcinoma espinocelular (CEC) anal, sendo geralmente reservada a tumores iniciais cuidadosamente selecionados. Entretanto, na prática clínica, a EL frequentemente é realizada em contextos mais amplos, especialmente em pacientes com grandes lesões perianais sem confirmação prévia de carcinoma invasivo, nos quais a incerteza diagnóstica pode influenciar a tomada de decisão.

**Objetivos:**

Avaliar os desfechos oncológicos após EL para CEC anal, com ênfase particular no papel das características da lesão, da incerteza diagnóstica e dos padrões de tratamento na prática de vida real.

**Métodos:**

Tratou-se de um estudo retrospectivo incluindo pacientes com CEC anal submetidos à EL como tratamento primário e acompanhados em um centro terciário oncológico entre 2010 e 2024. Foram coletados dados clínicos, anatomopatológicos e terapêuticos, incluindo características tumorais, status das margens, uso de terapia adjuvante e desfechos oncológicos.

**Resultados:**

Foram incluídos 20 pacientes com CEC anal invasivo. O tamanho tumoral médio foi de 3,24 cm (desvio padrão ±1,75 cm), com lesões de até 6,9 cm. Nenhum paciente recebeu quimiorradioterapia antes da EL. Apenas dois pacientes preencheram os critérios convencionais para EL (<2 cm, bem ou moderadamente diferenciados). No total, 14 pacientes (70%) necessitaram tratamento adicional após a EL, incluindo quimiorradioterapia ou amputação abdominoperineal. Entre os pacientes inicialmente conduzidos com vigilância, 44% apresentaram recorrência local, inclusive em casos com margens negativas.

**Conclusões:**

Os desfechos após a EL para CEC anal são fortemente influenciados pela seleção dos pacientes e pelo contexto clínico. Nesta coorte de vida real, a maioria dos pacientes que não se enquadravam nos critérios estabelecidos necessitou de tratamento adicional, o que ressalta as limitações da EL em lesões perianais extensas. Esses achados destacam a importância de uma avaliação pré-operatória criteriosa e da manutenção de um alto índice de suspeita para carcinoma invasivo, a fim de orientar a conduta inicial adequada.

## INTRODUCTION

 Anal squamous cell carcinoma (SCC) is an uncommon malignancy whose incidence has increased in recent decades, particularly among immunosuppressed populations^5^. Combined chemoradiotherapy (CRT) remains the standard of care, providing high rates of local control and survival^1,8,11,14^. 

 Local excision (LE) has a limited role and is generally reserved for carefully selected early-stage tumors, particularly small perianal lesions without sphincter involvement. When applied within these criteria, LE may allow avoidance of CRT-related toxicity while maintaining acceptable oncologic outcomes^1,11,14^. 

 In clinical practice, however, large perianal lesions often present a diagnostic challenge. These lesions may represent a spectrum of disease ranging from benign condyloma acuminatum to giant condyloma (Buschke-Löwenstein tumor) and invasive SCC. Previous studies have demonstrated that invasive carcinoma may be present in a substantial proportion of these lesions, supporting the concept of a biologic continuum rather than clearly distinct entities^3,4,13^. 

 As a result, LE is frequently performed with diagnostic or therapeutic intent in clinically ambiguous cases. In such scenarios, invasive carcinoma may only be identified on final pathology, and the procedure may not consistently adhere to oncologic principles. This diagnostic uncertainty complicates treatment selection and interpretation of outcomes. 

 The present study aimed to evaluate oncologic outcomes after LE for anal SCC, with particular emphasis on the role of lesion characteristics, diagnostic uncertainty, and treatment patterns in real-world practice. 

## METHODS

 A retrospective study was conducted including patients diagnosed with anal SCC who underwent LE and were followed at the Institution from March 2010 to January 2024. Cases were identified through queries of the institutional database using the International Classification of Diseases (ICD) code C21 for anal cancer. 

 Data collected included demographic characteristics, tumor size, histopathologic features (including differentiation, lymphovascular and perineural invasion), margin status, surgical details, use of adjuvant therapy, and clinical outcomes, including recurrence and survival. Postoperative complications and follow-up data were obtained from electronic medical records. 

 Given the retrospective nature of the study, the intent of LE (definitive treatment versus excisional biopsy) could not be consistently determined and was therefore interpreted in the context of lesion characteristics, margin status, and subsequent management. Only patients with histologically confirmed anal SCC were included. Patients who underwent local excision for benign lesions were not captured in this cohort. 

 The study was approved by the institutional review board, and the requirement for informed consent was waived due to the retrospective design and use of anonymized data. 

 Descriptive statistics were used to summarize patient, tumor, and treatment characteristics. Continuous variables were presented as mean ± standard deviation or median (range), as appropriate, and categorical variables as frequencies and percentages. Oncologic outcomes were analyzed descriptively. The study was approved by the Ethics Committee of the institution (CAAE 62915516.2.0000.0065). 

## RESULTS

 A total of 20 patients who underwent LE were included. Twelve (60%) were female, and 6 (30%) were living with the human immunodeficiency virus (HIV). The mean age at diagnosis was 58.6±9.2 years. Ten patients underwent surgery at the institution, while the remaining 10 were referred after initial treatment at other institutions. No patients received neoadjuvant chemotherapy or radiotherapy prior to LE. 

 With regard to surgical management, primary closure of the defect was performed in 8 patients (40%), healing by secondary intention was achieved in 3 (15%), and a local flap was used in 1 patient (5%). In 8 cases (40%), the closure technique was not documented. One patient developed an early postop erative complication — a partial flap dehiscence — which was successfully managed with antibiotics and local wound care. 

 Histopathologic analysis revealed a mean tumor size of 3.24±1.75 cm (range: 0.8–6.9 cm). Tumor staging was pT1 in 4 patients (20%), pT2 in 9 (45%), pT3 in 3 (15%), and pTx in 4 (20%), the latter due to specimen fragmentation. Most tumors were well or moderately differentiated (n=16; 80%), while 3 (15%) were poorly differentiated; tumor differentiation was not reported in 1 case (5%). Lymphovascular and/or perineural invasion was identified in 3 patients (17.6%). Positive surgical margins were observed in 9 patients (45%), and margin status was indeterminate in 4 (20%), primarily due to specimen fragmentation or incomplete documentation. 

 Adjuvant therapy was administered to 11 patients (55%), including combined chemoradiotherapy (CRT) in 7 cases and radiotherapy alone in 4. Among these patients, 2 developed recurrence and subsequently required abdominoperineal resection (APR). Of the 9 patients initially managed with surveillance, 4 (44%) developed local recurrence. Of these, 3 were treated with CRT and 1 underwent repeat LE; 1 patient treated with CRT ultimately required APR. 

 Overall, 14 patients (70%) required additional treatment following LE. During follow-up, 3 deaths were recorded: one due to metastatic disease, one in a patient with severe cardiac disease who developed cardiogenic shock following APR, and one of unknown cause. Two patients were lost to follow-up. Clinicopathologic characteristics and outcomes are summarized in Table 1. 

**Table 1:** Clinicopathologic characteristics and outcomes of patients treated with local excision for anal squamous cell carcinoma (n=20).

Patient	Age	Gender	Lesion size (cm)	pT	Tumor differentiation	Margins	Lymphovascular invasion	Perineural invasion	Additional treatment
1	53	F	3.0	2	Moderately differentiated	Positive	Negative	Negative	CRT
2	66	M	6.2	3	Moderately differentiated	Positive	Negative	Negative	RT
3	68	F	6.0	3	Moderately differentiated	Indeterminate	Not reported	Not reported	CRT
4	68	F	Fragmented	x	Moderately differentiated	Positive	Negative	Negative	CRT, APR
5	51	F	6.9	3	Moderately differentiated	Positive	Negative	Negative	CRT
6	59	M	2.3	2	Not reported	Negative	Negative	Negative	None
7	52	F	1.9	1	Well differentiated	Negative	Negative	Negative	None
8	43	F	Fragmented	x	Moderately differentiated	Indeterminate	Not reported	Not reported	CRT, APR
9	82	F	0.8	1	Poorly differentiated	Positive	Negative	Negative	RT
10	47	M	3.0	2	Moderately differentiated	Positive	Negative	Negative	CRT
11	61	M	2.5	2	Well differentiated	Positive	Negative	Negative	CRT
12	59	M	Fragmented	x	Well differentiated	Indeterminate	Negative	Negative	RT
13	88	M	1.9	1	Poorly differentiated	Negative	Negative	Positive	None
14	47	F	3.2	2	Moderately differentiated	Negative	Positive	Positive	RT
15	58	M	2.2	2	Moderately differentiated	Negative	Negative	Negative	CRT
16	59	F	Fragmented	x	Moderately differentiated	Indeterminate	Positive	Negative	None
17	44	F	1.5	1	Moderately differentiated	Negative	Positive	Negative	Repeat LE
18	44	F	2.9	2	Moderately differentiated	Negative	Negative	Negative	None
19	55	M	3.5	2	Moderately differentiated	Positive	Negative	Negative	CRT
20	69	F	2.9	2	Poorly differentiated	Positive	Not reported	Not reported	CRT

pT: tumor staging; M: male; F: female; CRT: chemoradiotherapy; RT: radiotherapy; APR: abdominoperineal resection; LE: local excision.

## DISCUSSION

 The findings demonstrate that outcomes following LE for anal SCC are strongly influenced by patient selection and the clinical context in which the procedure is performed. In this cohort, a substantial proportion of patients required additional therapy (70%), including CRT or APR, underscoring the limitations of LE when applied outside well-established indications. 

**Figure 1:**
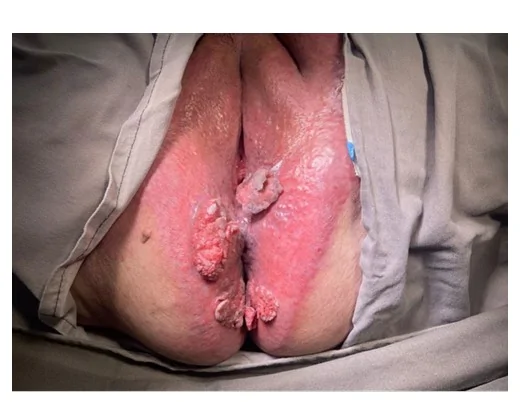
Large perianal condylomatous lesion with invasive carcinoma on final histopathology.

 CRT has been the standard treatment for most anal SCC since the 1980s, achieving high rates of local control and survival. Current guidelines reserve LE for highly selected cases, including superficially invasive disease or small perianal tumors (T1–T2N0) without sphincter involvement^1,8,11,14^. Nevertheless, LE continues to be performed in broader clinical contexts, particularly in larger perianal lesions lacking preoperative histopathologic confirmation of invasive cancer, reflecting variability in real-world practice (Figure 1). 

 A key finding of the study is the presence of relatively large lesions, with tumor sizes reaching up to 6.9 cm — well beyond conventional criteria for definitive LE. This observation aligns with prior descriptions of large perianal lesions representing a spectrum ranging from condyloma acuminatum to giant condyloma (Buschke-Löwenstein tumor) and invasive carcinoma. These entities share overlapping clinical and histologic features, and invasive SCC has been reported in a substantial proportion of giant condylomas, supporting the concept of a biologic continuum rather than clearly distinct categories^3,4,13^. 

 Within this framework, LE in this cohort likely reflects diagnostic uncertainty rather than a deliberate oncologic strategy. Large perianal lesions may be difficult to distinguish from benign conditions, and invasive carcinoma is often identified only on final pathology. As a result, procedures may not consistently adhere to oncologic principles, including adequate margins, proper specimen handling, and accurate staging. 

 Previous studies have reported favorable outcomes for LE in carefully selected early-stage disease. Chai et al., in a population-based cohort of 2,243 patients, demonstrated increased use of LE without a corresponding decrease in overall survival^2^. Similarly, a systematic review by Portale et al., including 3,323 patients, found comparable overall survival between LE and CRT in selected populations^10^. 

 For larger perianal lesions, particularly those within the spectrum of giant condyloma acuminatum, management remains heterogeneous and is largely based on limited evidence from case series. These lesions are characterized by high recurrence rates and a substantial risk of harboring or developing invasive SCC, reinforcing the concept of a biologic continuum rather than distinct entities. A variety of treatment strategies, including LE, topical therapies, and CRT, have been reported with variable outcomes, highlighting the lack of standardized management and the importance of careful evaluation and follow-up^13^. 

 In this series, only two patients met conventional criteria for LE (<2 cm, well or moderately differentiated tumors). One was managed with surveillance and remained recurrence-free, while the other developed a local recurrence requiring repeat excision without further treatment. Most of the remaining patients had additional adverse prognostic factors, such as poor differentiation and positive margin status, both of which have been associated with an increased risk of local recurrence^9^. Interestingly, some series have reported that tumor size may not be an independent risk factor for local recurrence^12^. Most patients in this cohort required additional treatment, including two who ultimately underwent APR, underscoring the potential severity of these cases. 

 The potential benefits of avoiding CRT with LE must be balanced against the risks of undertreatment. While CRT is associated with significant toxicity^6,7^, it remains a highly effective therapy. Ongoing prospective studies, such as the ACT3 trial, may further clarify the role of LE in early-stage anal SCC through risk-adapted strategies following excision. 

 Taken together, these findings have important practical implications for the evaluation and management of large perianal lesions. The results suggest that, in this setting, diagnostic uncertainty plays a central role in treatment decision-making and may lead to the use of LE outside conventional oncologic indications. When suspicion for invasive disease exists, targeted or multiple biopsies may improve diagnostic accuracy, although sampling error remains a limitation, and local staging with magnetic resonance imaging may provide additional value in selected cases. Importantly, outcomes following LE appear to be driven not only by the technique itself, but also by challenges in patient selection and preoperative diagnosis, underscoring the need for careful evaluation to optimize initial treatment and avoid delays in definitive therapy. 

 This study has several limitations, including its retrospective design, small sample size, and heterogeneous population. Anal SCC is a relatively rare malignancy, and most patients are managed with definitive CRT, which further limits the availability of patients undergoing LE. The patient profile in this cohort likely reflects referral patterns and case complexity typical of a tertiary cancer center, which may limit the generalizability of the findings. Nonetheless, these limitations reflect real-world practice and highlight the complexity of patient selection and management of large perianal lesions. 

## CONCLUSIONS

 The findings demonstrate that outcomes following LE for anal SCC are strongly influenced by patient selection and clinical context. In this real-world cohort, most patients outside established criteria for LE required additional treatment, underscoring the complexity of managing large perianal lesions and the limitations of LE in this setting. These results support a high index of suspicion for invasive carcinoma in large lesions and emphasize the importance of careful preoperative evaluation to guide optimal initial management. 

## Data Availability

The datasets generated and/or analyzed during the current study are available from the corresponding author upon reasonable request.
